# P-438. T-cell Immune Exhaustion and the Atherogenic Index of Plasma in People Living with HIV on Antiretroviral Therapy

**DOI:** 10.1093/ofid/ofae631.638

**Published:** 2025-01-29

**Authors:** Christian Francisco, Ana Joy Padua

**Affiliations:** National Institutes of Health, University of the Philippines Manila, Manila, National Capital Region, Philippines; College of Medicine, University of the Philippines Manila, Manila, National Capital Region, Philippines

## Abstract

**Background:**

The atherogenic index of plasma (AIP), an index that correlates with atherosclerosis, has been reported to be abnormal among people with human immunodeficiency virus (PWH). Few subclinical markers of atherosclerosis were linked to T-cell immune exhaustion. However, no study has explored the relationship of AIP to the different immune exhaustion markers. We compared the AIP and immunologic parameters of PWH on antiretroviral therapy (ART) to that of people without HIV (PWoH). Correlation of different clinical, laboratory and immunologic parameters with AIP in PWH were investigated.

**Table 1.**
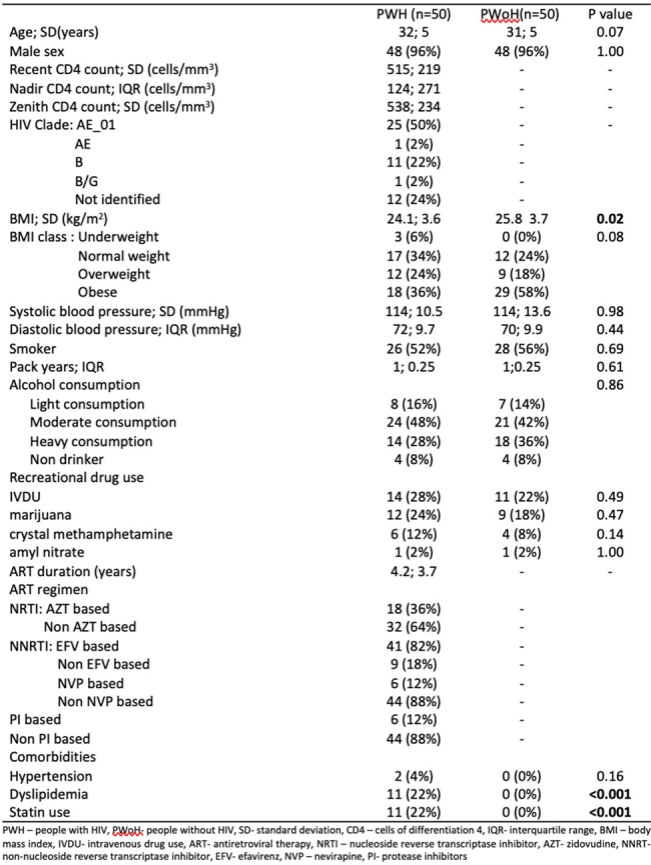
Demographic and clinical characteristics of study participants (N=100)

**Methods:**

This is a cross-sectional study conducted from March 2018 to February 2019 in a treatment hub in Manila, Philippines. We recruited 50 virally suppressed PWH on ART and 50 age- and sex- matched PWoH. Demographic and clinical data were recorded. Fasting blood samples were collected for complete blood count, blood glucose, and lipid profile. Peripheral blood monocytes were isolated and analyzed for immune exhaustion markers in T-cells and monocytes (LAG-3, PD-1, TIGIT+, TIM-3) via flow cytometry. AIP was computed as log (trigylyceride/high density lipoprotein-c). Abnormal AIP was defined as values in the medium (0.1-0.24) and high-risk ( >0.24) categories. Pearson correlation was performed to determine linear correlation between clinical, laboratory and immunologic factors and AIP.

**Table 2.**
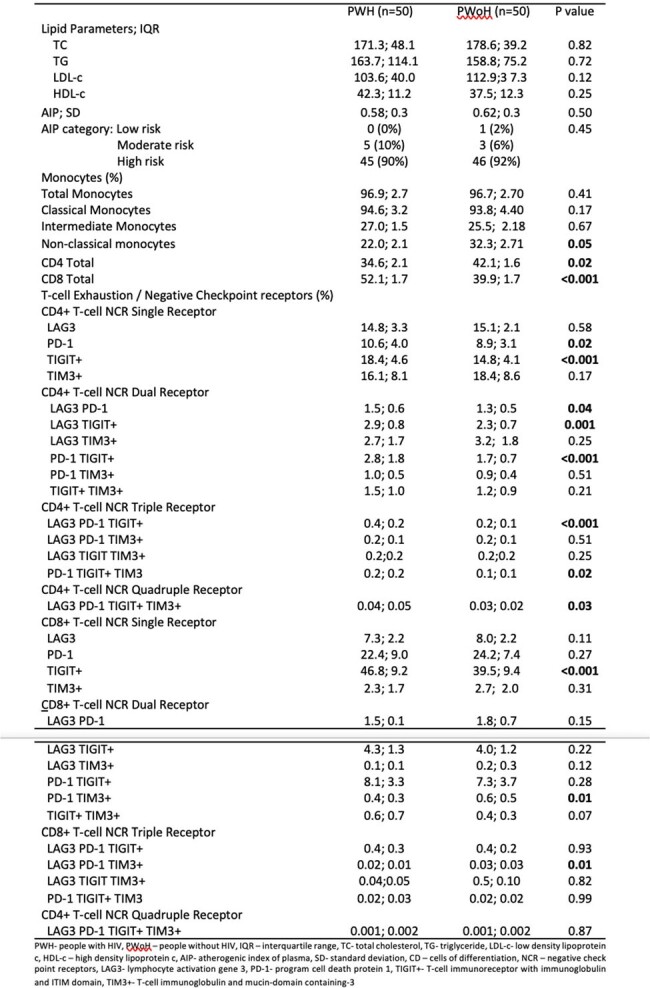
Laboratory and immunologic parameters of study participants (N=100)

**Results:**

Majority of the participants were male (96%) with mean age of 31. Among PWH, the mean recent CD4+ count is at 515 cells/mm3. Median ART exposure is 4 years. Majority were on efavirenz based ART (41, 82%). Few were on protease inhibitors (6, 12%). All PWH had abnormal AIP. There is no difference in AIP between PWH and PWoH (0.5; 0.3 vs 0.62; 0.3, p 0.5). Recent CD4+ (R 0.32, p 0.02), nadir Cd4+ (R 0.31, p 0.03) and zenith CD4+ (R 0.30, p 0.04) were positively correlated with AIP in PWH. Only dual expression of PD-1 TIGIT+ CD8+ T-cells showed positive correlation with AIP (R 0.20, p 0.04).

**Figure 1.**
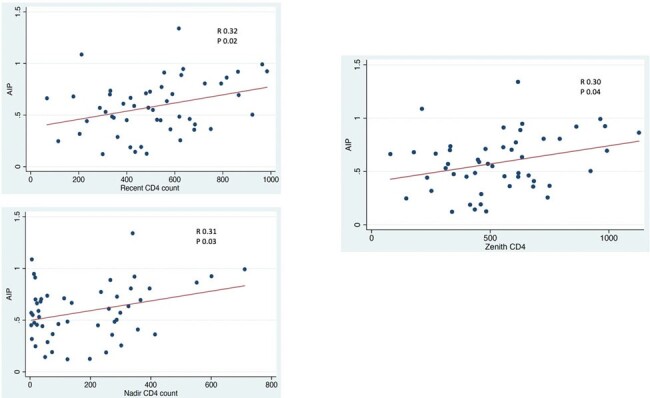
Correlation of AIP levels with CD4+ count

**Conclusion:**

AIP in PWH is comparable to that of PWoH. The correlation of AIP to PD-1 TIGIT+ CD8+ T-cells supports the role of T-cell immune exhaustion in the pathogenesis of dyslipidemia and atherosclerosis. Strategies to decrease immune exhaustion can possibly prevent or delay metabolic complications in PWH.

Correlation of AIP levels with frequency expression of PD-1 TIGIT+ on CD8+ T-cells

**Figure:**
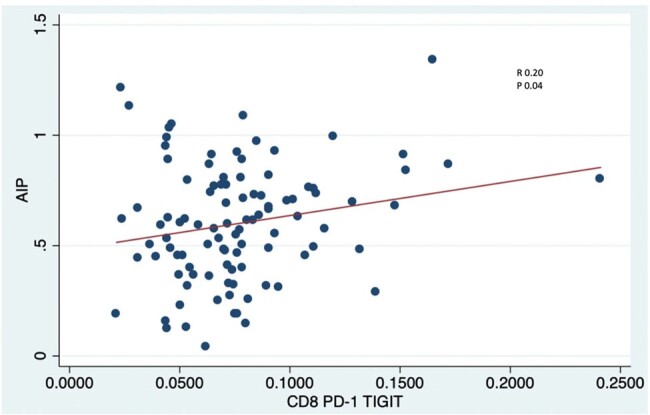

**Disclosures:**

**All Authors**: No reported disclosures

